# Informed consent for surgery on neck of femur fractures: A multi-loop clinical audit

**DOI:** 10.1016/j.amsu.2020.03.008

**Published:** 2020-04-08

**Authors:** Rohi Shah, Sharan Sambhwani, Awf Al-Shahwani, Christos Plakogiannis

**Affiliations:** Trauma and Orthopaedics Department, Kettering General Hospital, Rothwell Road, Kettering, NN16 8UZ, UK

**Keywords:** British orthopaedic association, Audit cycle, Informed consent, Neck of femur fractures, Montgomery case

## Abstract

**Background:**

The Montgomery case in 2015 resulted in a pivotal change in practice, leading to a patient-centric approach for informed consent. Neck of femur (NOF) fractures are associated with a high rates perioperative morbidity and mortality. Using guidelines highlighted by the British Orthopaedic Association we performed a multi-loop audit within our department to assess the adequacy of informed consent for NOF fractures.

**Methods:**

Two prior cycles had been performed utilising a similar framework. Prior interventions included ward posters, verbal dissemination of information at Junior Doctor's (JD) induction and amendments to the JD handbook. For the latest audit loop, a retrospective analysis of 100 patients was performed. Risk were classified as common, less common, rare and ‘other’ non-classifiable risks. The adequacy of informed consent was evaluated by assessing the quality and accuracy of documentation in the signed Consent Form-1s for compos mentis patients.

**Results:**

Infection, bleeding risks, clots and anaesthetic risks were documented in all patients (100%). Areas of improvement included documentation of neurovascular injuries (98%), pain (75%) and altered wound healing (69%). There was no significant change in the documentation of failure of surgery (83%) and neurovascular injuries (98%). Poorly documented risk factors included mortality (21%), prosthetic dislocation (14%) and limb length discrepancy (6%).

**Conclusion:**

Following the latest cycle, the trust has now approved the use of 2 consent-specific stickers (for arthroplasty or fixation), amendable on a patient-to-patient basis. As part of the multi-loop process, the cycle will be repeated every year, in line with Junior Doctor rotations. Medical professionals have an ethical, moral and legal obligation to ensure they provide all information regarding surgical interventions to aid patients in making an informed decision.

## Highlights

• The Montgomery case in resulted in a pivotal change in practice, leading to a patient-centric approach for informed consent.
• Medical professionals have an ethical, moral and legal obligation to ensure all pertinent information is passed to patients.
• Medico-legally, in the absence of formal documentation it is assumed that the process of informed consent is inadequate.
• The Trust-approved ‘consent sickers’ for arthoplasty/fixation are compliant with BOA-endorsed guidelines.

## 1 Introduction

The Montgomery Case in 2015 highlighted inadequacies in informed consent, thus setting legal standards required to advice patients about their treatment options and give effect to their preferences. Medical professionals are duty bound, having an ethical, moral and legal obligation to ensure that they provide all pertinent information regarding risks and benefits associated with surgical procedures to aid patients in making an informed decision.

It is well known that neck of femur (NOF) fractures are associated with a high rates peri- and postoperative morbidity and mortality. Using British Orthopaedic Association (BOA)-endorsed guidelines via the website: www.orthoconsent.com [1], we performed a multi-loop audit within our department to assess the adequacy of informed consent for NOF fractures. The website allows clinicians free access to a bank of procedure specific, pre-written consent forms. The BOA suggests that this guidance should be used as a benchmark against which clinical practice can be evaluated. Consenting in the acute trauma setting is generally recognized as being suboptimal [2], with patients not necessarily been given the most appropriate information to make an informed decision about risks associated with surgery.

The consenting guidelines classifies risk factors according to their severity. Common risk factors (2–5%) include: pain, bleeding, and blood clots (including deep vein thrombosis and/or pulmonary emboli). Less common risk factors (1–2%) include: infection, altered leg length discrepancy and prosthetic dislocation. Rare risk factors (<1%) included hip stiffness, altered wound healing, neurovascular injuries, failure of surgery, and significant mortality risk. Additionally, as part of our audit profile, we assessed the inclusion of anaesthetic risks. Within our trust, other than verbal discussion of anaesthetic risks by the Anaesthetist pre-operatively, there is no specific signed documentation for the discussion of the associated anaesthetic risks.

Two intra-departmental audits were performed in 2015 and 2016 to assess the documentation of risk factors for surgery whilst consenting compos mentis patients undergoing surgical management of their fractures in theatre. Both audits revealed variability in the number of risk factors documented by Junior Doctors (JD). It was unclear from the consent forms whether the undocumented risk factors were conveyed to patients and it was a case of not physically documenting them on the forms. Regardless, from a medico-legal perspective, in the absence of formal documentation it is assumed that the process of informed consent is inadequate. Interventions from these two audits were based on verbal and written dissemination of the results that included a printed copy of the ‘Risks associated with surgical interventions of NOF fractures’ that was displayed in the Orthopaedic Doctors' office. It is both easily accessible and serves as a reminder of mandatory documentation in Consent Form-1. Amendments have also been made to the JD induction handbook for Trauma and Orthopaedics at our local hospital which highlights all pertinent risk factors that must be clearly documented in all Consent Form-1 for NOF fractures.

The consenting process is usually performed by the on-call JD. Given the frequency of JD rotations within the Hospital, we performed a multi-loop audit to highlight any inadequacies in informed consent thus permitting early education and timely intervention.

## 2 Audit aims

The aim of this audit was to assess the quality and accuracy of the documentation of risks associated with surgery whilst consenting patients in preparation for surgical management of their NOF fractures.

## 3 Audit standards

The department standards were set at a 100% documentation of the common, less common and rare risks in the Consent Form-1 of all patients admitted with NOF fractures undergoing surgical intervention.

## 4 Methods

**Fig. 1:**
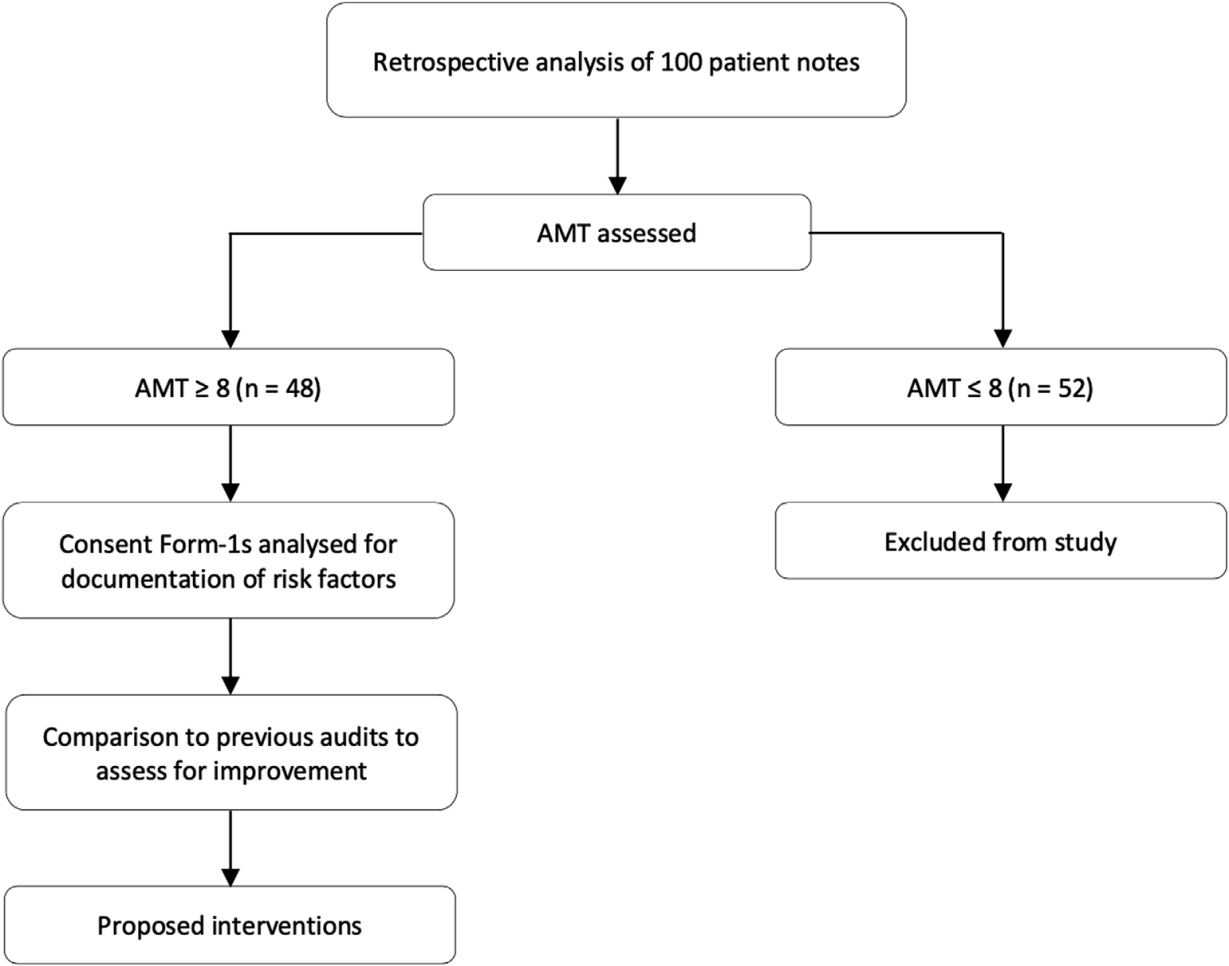
Methodology with patient selection criteria.

We performed a retrospective analysis of 2 cohorts of consecutive admissions, totaling 100 patients admitted with NOF fractures between September 2018 and September 2019. Of these, 48 patients who scored an Abbreviated Mental Test (AMT) Score of ≥8 on their initial assessment were deemed to have capacity to sign Consent Form-1 and were therefore included in the study. Those lacking capacity were omitted from the study as the decision to operate (or not) was made in the best interest of the patient by the admitting medical team (Fig. 1).

The risk factors for surgical interventions in NOF fractures were identified through direct documentation on the Consent Form-1s. It was assumed that in the absence of formal documentation, the risks factors were not discussed with the patient. Each form that was included in the study was reviewed to assess how many risks associated with surgical intervention had been included.

## 5 Results

Of the 100 patients, 48 patients were deemed to have capacity and included in the study. All 48 patients underwent surgical management. The surgical procedures varied from fixation i.e. sliding hip screw, intramedullary nailing or cannulated screw fixation (n = 27) and arthroplasty i.e. hip hemiarthroplasty or total hip replacement (n = 21).

Risks were once again classified according to their severity.

• Common risks (2–5%) included: pain, bleeding, and blood clots (including deep vein thrombosis and/or pulmonary emboli).
• Less common risks (1–2%) included: infection, altered leg length discrepancy and prosthetic dislocation (arthroplasty group).
• Rare (<1%) included hip stiffness, altered wound healing, neurovascular injuries, Non/mal-union (fixation group) and significant mortality risk.
• Other risks (non-classifiable): failure of surgery and anaesthetic risks.

**Fig. 2:**
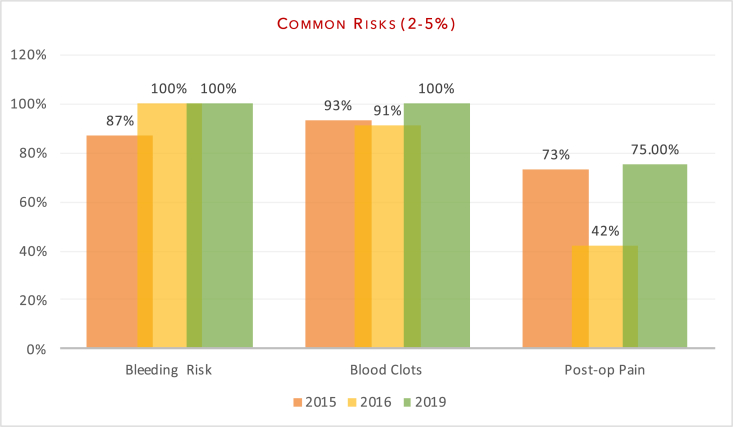
Documentation of common risks.

**Fig. 3:**
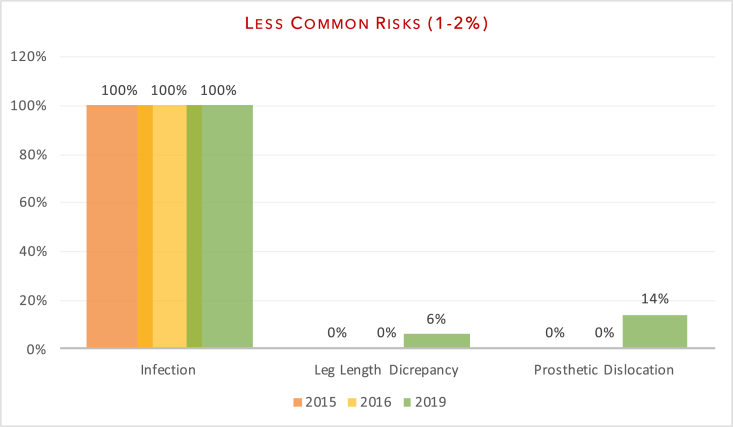
Documentation of less common risks.

**Fig. 4:**
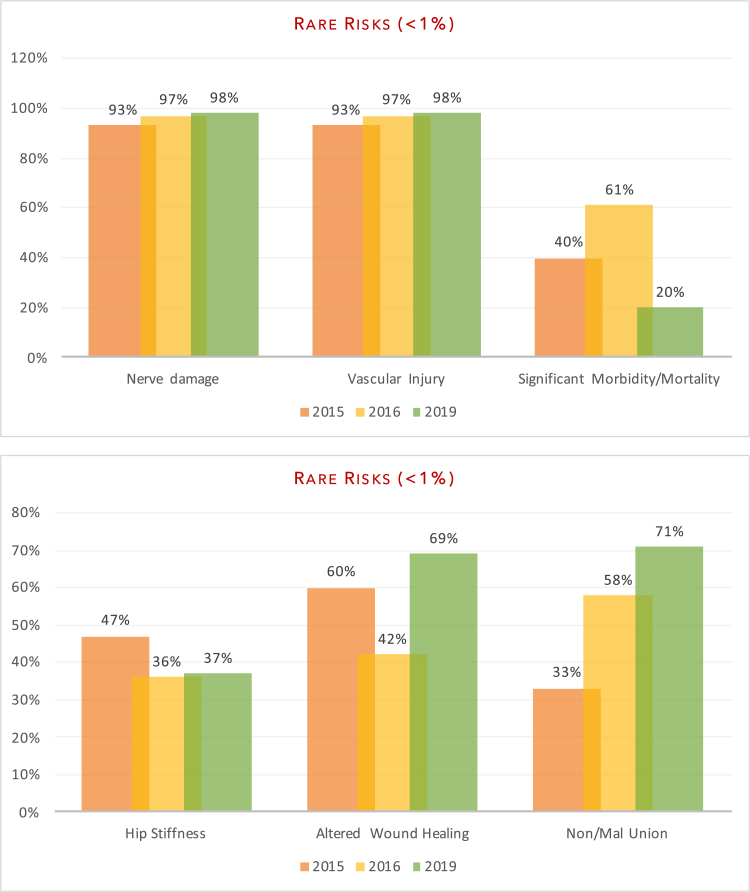
aDocumentation of Rare Risks Fig. 4b: Documentation of Rare Risks.

**Fig. 5:**
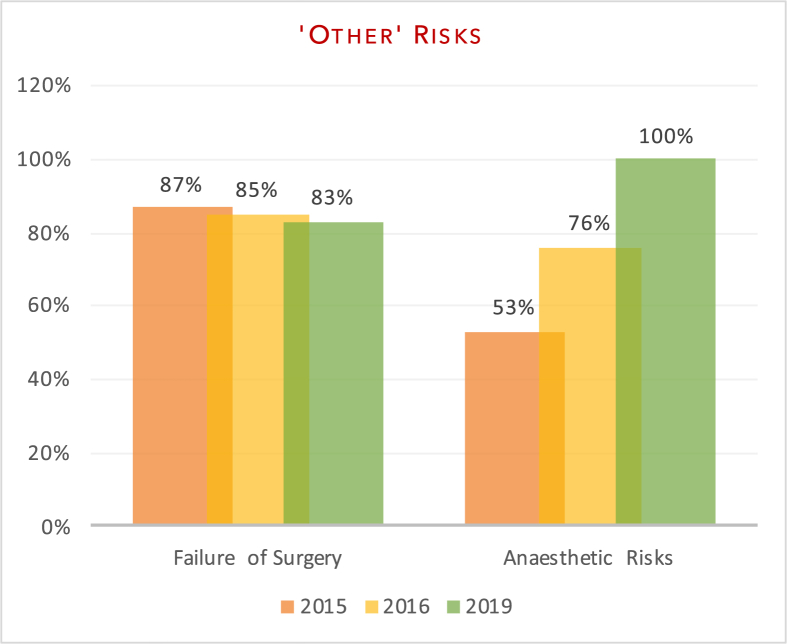
Documentation of additional non-classified risks.

The variation in documentation between the audits has been highlighted in Fig. 2, Fig. 3, Fig. 4, Fig. 5 below.

## 6 Discussion

Neck of femur fracturs are the most commonly reported fragility fractures in an older person with osteoporosis or osteopenia. The 2018 National Hip Fracture Database (NHFD) reported 65,958 admissions over the year [3]. Life expectancy is set to increase in the UK due to an ageing population, availability of better medical services and medical interventions. As such, this figure is likely to increase on a yearly basis. The projected rise in the number of hip fractures is estimated at 100,000 by 2033 [4].

The General Medical Council and the Royal College of Surgeons [5,6] require consent to be obtained for any surgical procedure. Whilst the healthcare profession proposing and performing the procedure is ultimately responsible for taking the patient's consent, this role can be delegated to a person appropriately trained, who has specific knowledge of the procedure and its risks [5]. Unfortunately, in most hospitals, this usually falls to the most Junior member of the team [7,8] who undertakes this process. Due to variability in experience, they may not necessarily be aware of all the risks the patient may be exposed to [2,7,8]. This no doubt leads to inadequacies and gaps in the process [9,10]. This highlights the importance of carrying out repeat audit loops that coincide with new Junior Doctors rotating into the department, with prior results being highlighted at their induction.

The law on informed consent has changed following a Supreme Court judgement in the case of Montgomery v Lanarkshire Health Board (2015), which now requires a Doctor to take ‘reasonable care to ensure that the patient is aware of any material risks involved in any recommended treatment, and of any reasonable alternative or variant treatments’. Compared to the previously accepted ‘Bolam Test’ where a Doctors conduct would be supported by a responsible body of medical opinion, informed consent has now shifted towards a patient-centric approach. Informed Consent is an ethical and legal prerequisite before any investigation, treatment or intervention of a patient. The philosophy behind this process treats patients as autonomous individuals who are presented with complete, evidence based information about the benefits and the risks of the proposed intervention to allow for a rational choice, free from duress [5,11].

The aim of our study was to evaluate department adherence to BOA-endorsed guidelines and to assess whether patients were truly making an ‘informed decision’ pertaining to surgery. Our re-audit once again demonstrated variability in the number of risk factors documented on the consent forms. In line with previous audits, all patients had documentation of infection risk (100% (2019) vs 100% (2016) vs 100% (2015)) and bleeding risk (100% vs 100% vs 87%). Areas of significant improvement included anaesthetic risks (100% vs 76% v 53%) and clots; inclusive of deep vein thrombosis and pulmonary emboli (100% vs 91% vs 93%). Documentation of neurovascular injuries also improved (98% vs 97% vs 93%) as did pain (75% vs 42% vs 73%) and altered wound healing, including scarring (69% vs 42% vs 60%). Of the subset of patients that underwent fixation (sliding hip screw, intramedullary nailing or cannulated screws), there was improvement in the documentation of non/mal-union (71% vs 58% vs 33%) as a post-operative risk. There was no significant change in the documentation of failure of surgery (83% vs 85% vs 87%). Poorly documented risks included significant morbidity/mortality risk (21% vs 61% vs 40%), prosthetic dislocation (for the arthroplasty group) (14% vs 0% vs 0%) and leg-length discrepancy (6% vs 0% vs 0%).

Despite improvements in many areas, the department is failing to achieve the 100% recommended target. A common misconception that risk factors deemed ‘generic’ and ‘obvious’ for any form of surgical intervention such pain, post-operative stiffness and scarring can be discussed via verbal consent rather than documented is incorrect. From a medico-legal perspective, as well as for patient understanding, these need to be discussed in detail and clearly documented. Procedure specific risk factors such as prosthetic dislocation for hip hemiarthroplasties or non-union, malunion or avascular necrosis in fixative surgery should also be highlighted to patients. Our study highlights inadequacies in this particular aspect of informed consent. Additionally, post-procedure complications that may lead to significant morbidity or mortality (deep vein thrombosis, pulmonary emboli, neurovascular injuries) should be discussed in detail with patients. Documentation of morbidity/mortality is well debated, and often depends on the clinical situation i.e. a ‘younger’ fitter patient undergoing surgical fixation would have a much lower risk of a significant cardiovascular event. However, as the risk still exists, no matter how low, and as such, it should still be discussed whilst consenting the patient. This also gives Doctors an opportunity to discuss resuscitation status with the patient and thus having a pre-operative escalation plan.

The latest audit loop also highlighted the inappropriate use of medical abbreviations (DVT for deep vein thrombosis, PE for pulmonary embolism, N/V damage for neurovascular damage). Whilst its use is self-explanatory for medical professionals, these words are often overlooked and misunderstood by patients. Therefore, every effort should be made to replace medical abbreviations with terms all patients should be able to comprehend such as ‘risk of blood clots in the leg or to the lungs’ to allow patients to re-read, understand and ask any pertinent questions relating to their surgery.

### 6.1 Interventions & recommendations

The following interventions have been made following the results of the latest audit:

• A printed A4 sheet highlighting ‘Risk factors associated with surgical interventions of NOF fractures’ is currently displayed on the wards and in the Orthopaedic Doctors' office. The document highlights the audit results. It is easily accessible and serves as a reminder of mandatory documentation in Consent Form-1 (Appendix A).
• The results of the audit have been conveyed to each member of the Trauma and Orthopaedics team during the departmental audit presentation session.
• Amendments have been made to the Junior Doctors Induction Handbook for Trauma and Orthopaedics at our Hospital to include a page on risk documentation standards for all patients undergoing surgical stabilization of NOF fractures. This is further discussed during departmental induction for all new Doctors rotating into the department.
• The trust has approved the use 2 consent-specific stickers (arthroplasty/fixation). This contains all necessary information pertaining to the patient's surgery and figures may be amended on a patient to patient basis. (Appendix B).
• As per trust policy, a copy of the consent form (which would include the consent-specific sticker) will handed over to the patient pre-operatively to re-read and understand as well as give them an opportunity to clarify any questions at a later stage.
• With the rotation of Junior Doctors every 6 months to a year, we recommended the timing of repeat audit loops to coincide with this change-over in Doctors. This will allow for early education and timely intervention to occur.

## 7 Conclusion

Despite areas of notable improvements, the department is yet to achieve the recommended standard as per the BOA-endorsed guidelines. In addition to repeating prior interventions i.e. verbal and written dissemination of information and amendments to the JD induction handbook, the trust has approved the use 2 consent-specific stickers (arthroplasty/fixation) which may be amended on a patient to patient basis. This particular intervention ensures that there is appropriate information dissemination to all patients undergoing surgery.

As part of the multi-loop process, the audit cycle will be repeated every year, in line with Junior Doctor rotations.

We strongly believe that medical professionals have an ethical, moral and legal obligation to ensure they provide all information regarding surgical interventions to aid patients in making an informed decision.

## Ethical approval

This study was done as part of a local quality improvement/audit process and as such no ethical approval was required.

The study has been registered with the local audit department.

## Sources of funding

This research received no funding.

## Author contribution

All authors contributed equally and were involved in the study and preparation of the manuscript.

## Research registration number

Registered to local audit department.

Name of the registry: Research Registry.

Unique Identifying number or registration ID: researchregistry5414.

Hyperlink to the registration (must be publicly accessible):

https://www.researchregistry.com/browse-the-registry#home/?view_2_search=researchregistry5414&view_2_page=1.

## Guarantor

Rohi Shah (Lead Author).

## Provenance and peer review

Not commissioned, externally peer reviewed.

## Declaration of competing interest

None of the authors have any conflicts of interest to declare.

## Footnotes

Appendix Supplementary data to this article can be found online at https://doi.org/10.1016/j.amsu.2020.03.008.
