# Supplementary material for: Informed consent for surgery on neck of femur fractures: A multi-loop clinical audit
Source: Ann Med Surg (Lond). 2020 Apr 8;54:26–31. doi: 10.1016/j.amsu.2020.03.008 (PMC7242500; doi:10.1016/j.amsu.2020.03.008)
Supplement: Appendix A [file mmc1.pdf]

Appendix A

Informed Consent For Surgery On Neck Of Femur Fracture: A Multi-Loop Re-Audit

Mr. Sharan Sambhwani | Mr. Rohi Shah | Mr. Awf Al-Shawani | Mr. Christos Plakogiannis

All patients deemed compos mentis (AMT ≥ 8) undergoing surgery for neck of femur fractures should have verbal and documented discussion of risk factors on their Consent Form-1s. The British Orthopaedic Association (BOA) classifies risks factors according to their severity:

Common Risks (2-5%):

Pain, bleeding, and blood clots (including deep vein thrombosis and/or pulmonary emboli).

Less Common Risks (1-2%):

Infection, altered leg length discrepancy and prosthetic dislocation,

Rare Risks (<1%):

Neurovascular injuries, significant mortality risk, Hip stiffness, altered wound healing, non/mal-union

Other Risks

Failure of surgery/anaesthetic risks

We have an ethical, moral and legal obligation to ensure we provide the information regarding the benefits and risks associated with surgical procedures to aid patients in making a decision. Results of the latest audit have been highlighted below:

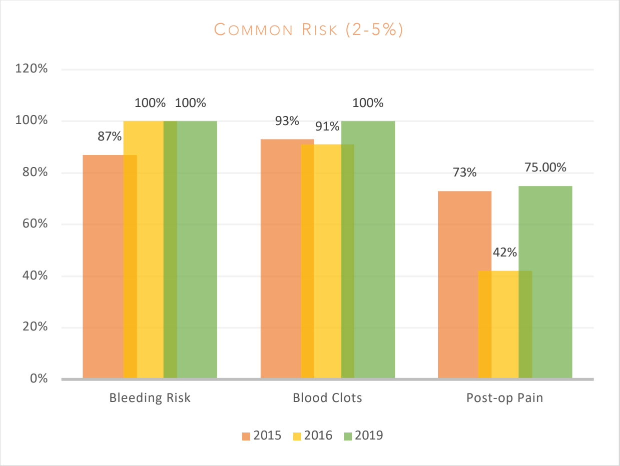

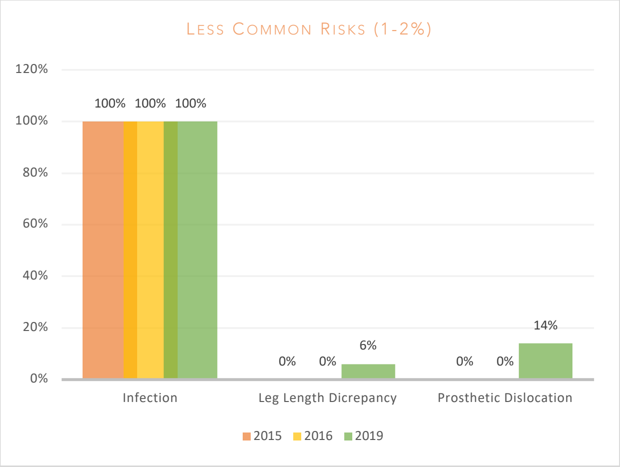

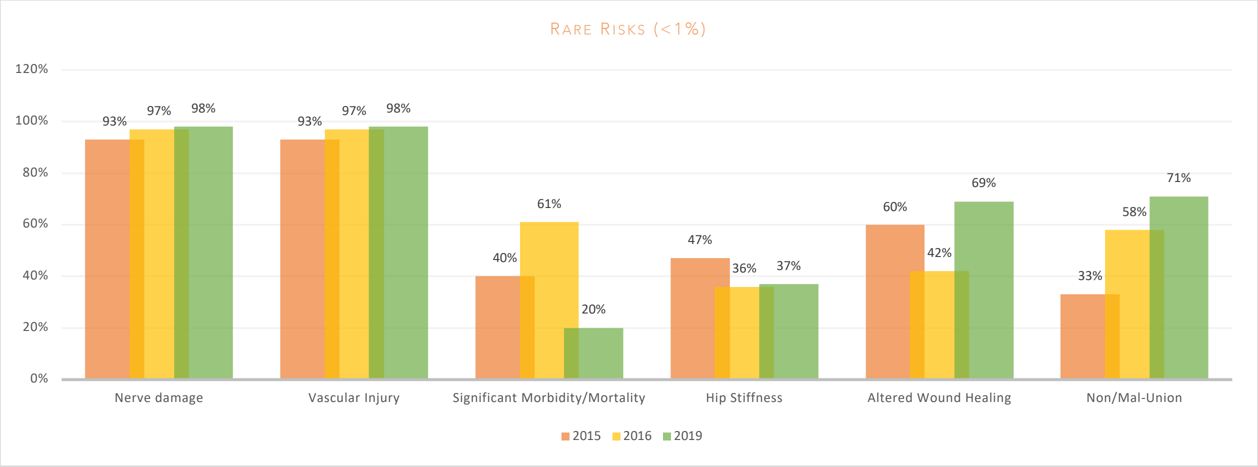

'Other' Risks

Failure of surgery (87% (2015) vs 85% (2016) vs 83% (2019))
Anaesthetic risk (53% (2015) vs 76% (2016) vs 100% (2019))

December 2019
