# Supplementary material for: Informed consent for surgery on neck of femur fractures: A multi-loop clinical audit
Source: Ann Med Surg (Lond). 2020 Apr 8;54:26–31. doi: 10.1016/j.amsu.2020.03.008 (PMC7242500; doi:10.1016/j.amsu.2020.03.008)
Supplement: Appendix B [file mmc2.pdf]

Appendix B

FIXATION

**Common Risks (2-5%):** Pain, bleeding, and blood clots (including deep vein thrombosis and/or pulmonary emboli).
**Less Common Risks (1-2%):** Infection, altered leg length discrepancy and anaesthetic risks
**Rare (<1%)** Hip stiffness, altered wound healing, neurovascular injuries, failure of surgery, non/mal-union, significant morbidity/mortality risks

ARTHROPLASTY

**Common Risks (2-5%):** Pain, bleeding, and blood clots (including deep vein thrombosis and/or pulmonary emboli).
**Less Common Risks (1-2%):** Infection, altered leg length discrepancy, prosthetic dislocation and anaesthetic risks
**Rare (<1%)** Hip stiffness, altered wound healing, neurovascular injuries, failure of surgery, significant morbidity/mortality risks
